# Learning from Host-Defense Peptides: Cationic, Amphipathic Peptoids with Potent Anticancer Activity

**DOI:** 10.1371/journal.pone.0090397

**Published:** 2014-02-28

**Authors:** Wei Huang, Jiwon Seo, Stephen B. Willingham, Ann M. Czyzewski, Mark L. Gonzalgo, Irving L. Weissman, Annelise E. Barron

**Affiliations:** 1 Department of Bioengineering, Stanford University, Palo Alto, California, United States of America; 2 Institute for Stem Cell Biology and Regenerative Medicine, Stanford University, Palo Alto, California, United States of America; 3 Department of Urology, Stanford University, Palo Alto, California, United States of America; 4 Department of Pathology, Stanford University, Palo Alto, California, United States of America; 5 Division of Liberal Arts and Sciences and Department of Chemistry, Gwangju Institute of Science and Technology, Gwangju, Republic of Korea; 6 Department of Chemical and Biological Engineering, Northwestern University, Evanston, Illinois, United States of America; Univ of Bradford, United Kingdom

## Abstract

Cationic, amphipathic host defense peptides represent a promising group of agents to be developed for anticancer applications. Poly-*N*-substituted glycines, or peptoids, are a class of biostable, peptidomimetic scaffold that can display a great diversity of side chains in highly tunable sequences via facile solid-phase synthesis. Herein, we present a library of anti-proliferative peptoids that mimics the cationic, amphipathic structural feature of the host defense peptides and explore the relationships between the structure, anticancer activity and selectivity of these peptoids. Several peptoids are found to be potent against a broad range of cancer cell lines at low-micromolar concentrations including cancer cells with multidrug resistance (MDR), causing cytotoxicity in a concentration-dependent manner. They can penetrate into cells, but their cytotoxicity primarily involves plasma membrane perturbations. Furthermore, peptoid **1**, the most potent peptoid synthesized, significantly inhibited tumor growth in a human breast cancer xenotransplantation model without any noticeable acute adverse effects in mice. Taken together, our work provided important structural information for designing host defense peptides or their mimics for anticancer applications. Several cationic, amphipathic peptoids are very attractive for further development due to their high solubility, stability against protease degradation, their broad, potent cytotoxicity against cancer cells and their ability to overcome multidrug resistance.

## Introduction

Chemotherapy remains a must-have treatment for many cancer patients, especially in those with advanced diseases [1]. Classic chemotherapeutical agents typically target the rapid proliferation of tumor cells by interrupting the synthesis or the functions of DNA, RNA or proteins. The lack of specificity of these drugs usually leads to many severe side effects. Moreover, it is very common for patients to develop multidrug resistance (MDR) through the efflux of drugs from cancer cells and become unresponsive to multiple chemotherapeutics [2]. In order to overcome drug resistance and provide more effective treatment, new molecular platforms with anti-proliferative activity employing a novel mechanism of action have been actively investigated.

Antimicrobial peptides, also known as host-defense peptides, represent a class of naturally occurring compounds that have recently been explored for their anticancer activity [3], [4], [5], [6], [7]. Natural products have been playing an important role in developing chemotherapeutics with a substantial amount of anticancer agents in use being either natural or derived from natural products from various sources [8]. Antimicrobial peptides are evolutionarily ancient weapons found throughout the animal and plant kingdoms [9]. A hallmark of this class is that the molecule can adopt a structure in which clusters of cationic and hydrophobic residues are spatially organized in discrete sectors, and this cationic, amphipathic structural feature is critical for their activity and selectivity [10]. Most host-defense peptides are believed to be membranolytic, with cationic residues selecting for anionic cellular membranes via electrostatic interactions and hydrophobic regions responsible for membrane permeation and disruption [11]. Magainin 2 and its analogs were first found in 1993 to display selective cytoxicity towards carcinoma cells *in vitro* and were proven to be as effective as doxorubicin *in vivo* via intraperitoneal delivery in ovarian cancer mouse models [12]. Over the last two decades, a growing number of studies have shown that some cationic, amphipathic peptides, including both natural host defense peptides and synthetic antimicrobial peptides, exhibit a broad spectrum of cytotoxic activity against cancer cells and are effective in reducing tumor burdens in several cancer animal models [3], [4], [5], [6], [7]. The selectivity of these peptides towards cancer cells is not well understood and is hypothesized to result from some altered membrane properties of cancer cells compared to normal tissue cells, e.g., more negative charges on outer membrane leaflets, more microvilli, higher transmembrane potentials, or higher membrane fluidity [3], [4], [5], [6], [7].

This class of cationic, amphipathic peptides possesses many features ideal for anticancer applications, including 1) high water solubility, 2) broad, potent cytotoxicity against cancer cells, and 3) the ability to overcome multidrug resistance developed in cancer cells [12], [13], [14]. However, the clinical use of peptide-based drugs has been limited due to their rapid degradation and clearance *in vivo*. Non-natural peptidomimetics can circumvent this proteolytic sensitivity while retaining the beneficial features of peptides [15]. A group of stable diastereomeric lytic peptides (containing both D- and L-forms of lysines and leucines) was developed based on antimicrobial model amphipathic peptides and investigated for anticancer applications [16], [17], [18]. Poly-*N*-substituted glycines, or peptoids, comprise another class of protease-resistant peptidomimetics, with side chains attached to the backbone nitrogen rather than to the α-carbon as in peptides [19], [20]. Peptoids can be readily synthesized on solid phase in a sequence-specific manner on an automated peptide synthesizer. Virtually any desired chemical functionality available as a primary amine can be incorporated into peptoids via a submonomer method, which provides great chemical “design diversity” in peptoid sequences [20].

Peptoids provide an ideal molecular platform to mimic the cationic, amphipathic structural feature of host-defense peptides. Though lacking backbone chirality and intrachain hydrogen bonding, peptoids can readily form helical structures with a periodic incorporation of bulky, α-chiral side chains, giving rise to polyproline type-I-like helices with approximately three residues per turn and a helical pitch of 6.0 – 6.7 Å [20], [21], [22]. This threefold periodicity of peptoids allows the cationic, amphipathic structure to be easily recapitulated in three-faced helices, simply using peptoids comprising trimer repeats, (X-Y-Z)_n_, which will display X, Y, and Z residues on separate faces. Accordingly, our group developed antimicrobial peptoids by mimicking magainin-2 [23]. A library of antimicrobial peptoids that typically adopt cationic, amphipathic helical structures was synthesized and studied for the relationships between their structures and their antimicrobial activity and selectivity [24], [25].

Here, inspired by anticancer peptides, we developed a new library of cationic, amphipathic peptoids and screened their anticancer activity and selectivity *in vitro*. We demonstrated for the first time that cationic, amphipathic peptoids can exhibit potent, fast cytotoxicity at low micromolar concentrations to a broad range of human cancer cell lines, and some peptoids were developed to show modest *in vitro* selectivity towards cancer cells. Moreover, actions of these peptoids were not influenced by multidrug resistance, killing primarily via plasma membrane disruptions. Finally, *in vivo* efficacy of the most potent peptoid derivative was validated in a preliminary study using a breast cancer xenotransplantation model established with human patient tumor cells.

## Materials and Methods

### Peptoid synthesis and purification

Peptoids were synthesized using an ABI 433A peptide synthesizer (Applied Biosystems, Inc.) on Rink amide MBHA resin (EMD Biosciences, Gibbstown, NJ) using the submonomer protocol [20], [24]. Briefly, the amine on the nascent chain is bromoacetylated or chloroacetylated followed by S_N_2 displacement of bromide or chloride by a primary amine to form the side chain. Resin-bound peptoids were then exposed to a mixture of trifluoroacetic acid (TFA): triisopropylsilane: water (95∶2.5∶2.5, volume ratio) for 10 minutes to cleave peptoids from the resin. Crude peptoids were purified by reversed-phase high performance liquid chromatography (RP-HPLC) (Waters Corporation) using a C18 column and a linear acetonitrile/water gradient. A final purity >95% as measured by analytical RP-HPLC (Waters Corporation) was achieved, and the identity of each peptoid was confirmed using electrospray ionization mass spectrometry (ESI/MS). Pexiganan was synthesized by standard Fmoc chemistry on an ABI 433A peptide synthesizer (EMD Biosciences). Unless indicated otherwise, all reagents were purchased from Sigma Aldrich (St. Louis, MO). Among the submonomers used, *N*spe was derived from (*S*)-*N*-(1-phenylethyl)amine; *N*pm from benzylamine (1-phenylethylamine); *N*His from histamine (2-[4-imidazolyl]ethylamine); *N*Leu from isobutylamine; *N*Lys from *N*-*tert*-butoxycarbonyl-1,4-butanediamine (CNH Technologies, MA). Guanidinylation of *N*Lys was carried out according to the reported procedure [26]. When *N*His was used in the peptoid sequence, chloroacetic acid was used [27]. 5(6)-Carboxyfluorescein was used to label the N-terminus of peptoid **1** [28].

### Cell cultures

MRC-5 were purchased from American Type Culture Collection (ATCC) and were grown in media suggested by ATCC supplemented with 10% FBS (Hyclone, US sources) and antibiotics (Sigma). MCF-7, MCF-7/TxT50 and OVCAR-3 (available in ATCC) were kindly provided by Professor Branimir Sikic's lab at Stanford University [35], and were cultured in McCoy's 5A media (GIBCO) with 10% FBS and antibiotics. Primary dermal fibroblasts were gifts from Lifeline Cell Technology and were cultured <6 passages according to the protocol Lifeline provides. All the cells were cultured in cell incubators (Thermo Scientific) at 37°C with 5% CO_2_.

### The MTS assay

This assay is a colorimetric method for determining the number of viable cells in proliferation. Aliquots of 100 µl media containing 1×10^4^ cells were distributed into each well of a 96-well plate (BD Falcon). The following day, when cell density reaches about ∼40% confluency, the cell media were removed and replaced with serial dilutions of peptoid stocks in culturing media. For peptoid dilutions, peptoid stocks were initially diluted in media at 100 µM and then diluted by half in series using a multichannel pipette, and maintained in 100 µl media for each concentration with triple repeats. After peptoid solutions were transferred onto cells in 96-well plates, cells were incubated at 37°C for certain time periods. Then 20 µl of the CellTiter 96 Aqueous Non-Radioactive cell proliferation assay (Promega) reagent which contains a tetrazolium compound, [3-(4,5-dimethylthiazol-2-yl)-5-(3-carboxymethoxyphenyl)-2-(4-sulfophenyl)-2H-tetrazolium, inner salt; MTS(a)], was added to each well and cells were further incubated for 2 h to metabolize. The absorbance of formazan products were measured at 490 nm in a microplate reader (Molecular Devices). Percentage of cell viability  =  (*A* -*A*_testblank_)/(*A*_control_ -*A*_blank_)×100, where *A* is the absorbance of the test well and *A*_control_ the average absorbance of wells with cells not treated with peptoids. *A*_testblank_ (media, MTS, and diluted peptoids) and *A*_blank_ (media and MTS) were background absorbances measured in the absence of cells.

### The Guava Viacount assay

This assay measures cell viability by using two DNA-binding dyes, one membrane-permeant, the other membrane-impermeant. Briefly, cells were plated and peptoids were diluted as described in MTS assays. After peptoid treatments, media supernatant with floating cells were collected. Cells were washed once, trypsinized and neutralized with previously collected media supernatant with floating cells. Cells were centrifuged at 1800 rpm for 3 min, and cell pellets were resuspended in 180 µl of fresh media. 20 µl of Guava Viacount reagents (Millipore) was added to each cell suspension, gently mixed and incubated at room temperature (protected from light) for 5 min before being submitted to the Guava Easycyte Plus flow cytometry system (Millipore) to measure the cellular fluorescent signals stained with dyes in Viacount reagents and to quantify cell viability.

### The LDH assay

This assay is used to measure the membrane integrity as a function of the amount of cytoplasmic LDH (lactic dehydrogenase) released into the medium. Experiments were carried out according to the protocol of the *In Vitro* Toxicology Assay Kit, Lactic Dehydrogenase (LDH) based (Sigma-Aldrich). Briefly, cells were plated as described before, and peptoids were diluted similarly but in culturing media without phenol red to reduce background signal. After peptoid treatments, media supernatant were collected and centrifuged to remove any cell debris, and analyzed for LDH activity in a 96-well plate using the kit, absorbance at 490 nm and 690 nm measured using a microplate reader. All the following absorbance difference =  *A*_490nm_ –*A*_690nm_. Percentage of LDH leakage  =  (*A* -*A*_testblank_)/(*A*_lysis_ -*A*_blank_)×100, where *A* is the average absorbance difference of the test wells and *A*_lysis_ the average absorbance difference of wells with cells treated with cell lysis solution provided by the kit for 45 min. *A*_testblank_ (media, diluted peptoids and LDH measuring reagents) and *A*_blank_ (media and LDH measuring reagents) were background absorbance differences measured in the absence of cells.

### Confocal imaging

MCF-7 cells seeded in 35 mm glass-bottom dishes (MatTek Corporation) were treated with 8 µM carboxyfluorescein-peptoid1 (CF-peptoid **1**) diluted in culturing media with 10% FBS for 1 h. Supernatants were removed, and cells were washed with PBS three times before being imaged via a Leica SP2 AOBS confocal laser scanning microscope.

### Peptoid evaluation in a human breast cancer xenograft mice model

80,000 cells from a dissociated second generation metastatic breast cancer tumor were suspended in Medium 199 containing 25% Matrix Matrigel (Becton Dickinson 354248) and injected into the mammary fat pad of 4–8 week old NOD.Cg-Prkdc^scid^ Il2rg^tm1Wjl^/SzJ (NSG) mice. After two weeks, 100 µl PBS containing 110 µM peptoid **1** or control peptide was injected into the mammary fat pad three times a week, roughly at a dose of 1 mg/kg. Tumor volumes were determined using direct caliper measurements following twelve weeks of treatment.

### Ethics Statement

This study was carried out in strict accordance with the recommendations in the Guide for the Care and Use of Laboratory Animals of the National Institutes of Health. The protocol was approved by Stanford University Institutional Animal Care and Use Committee (IACUC) (Protocol Number: 10725).

## Results

### Peptoid Design and *in vitro* Screening

**Figure 1:**
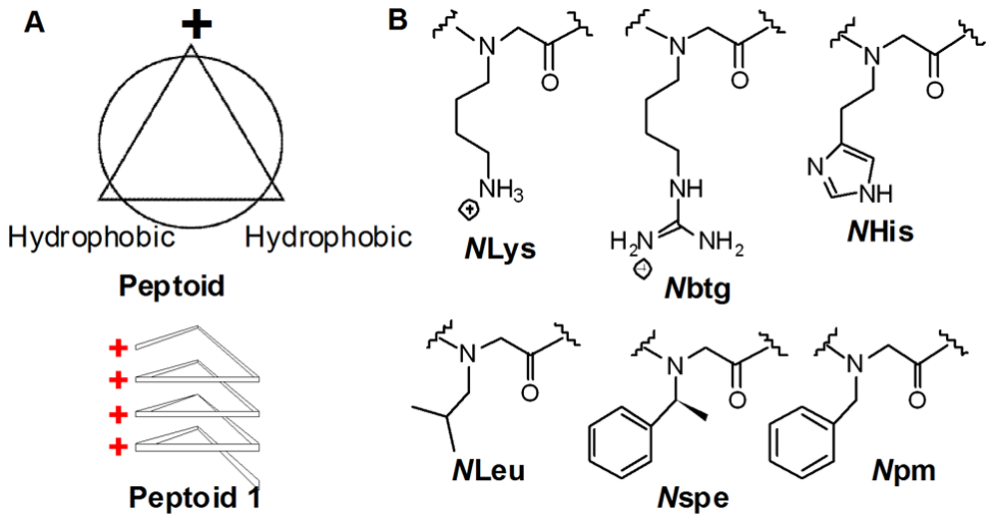
The cationic, amphipathic structure of peptoids and monomers. A, top-view of the cationic, amphipathic structure of peptoids (upper), and side-view of the helical structure of peptoid 1 (bottom); B, peptoid monomer side-chain structures with shorthand names.

Peptoids were utilized herein as a peptidomimetic scaffold to capture the cationic, amphipathic nature of anticancer peptides, as well as to improve molecular stability and to increase chemical diversity. The design of anticancer peptoids were derived from previous antimicrobial peptoids and were further optimized hererin to improve the activity and selectivity of peptoids against anionic membranes [23], [24], [25]. Peptoid **1**, [H-(*N*Lys-*N*spe-*N*spe)_4_-NH_2_], composed of *N*Lys (the peptoid analog of Lys) and *N*spe (Figure 1), is the most active antimicrobial dodecamer we had previously developed yet it had some minor hemolytic effects at high concentrations (Figure 1A). Several novel variants were designed based on peptoid **1** but with different charges, amphipathicity, length, and helicity to change their activity or selectivity. The sequences of these variants, the solvent composition at RP-HPLC elution as a relative measure of molecular hydrophobicity, molecular net charges and their charge-to-length ratio are summarized in Table 1. Variants marked with an asterisk were previously reported to possess antimicrobial activity [24], [25]. Pexiganan, a clinically-relevant peptide analog of magainin 2 which was developed for topical treatment for diabetic foot infection [29], and LL-37, the only known human cathelicidin which is a non-selective antimicrobial peptide [30], were also tested for comparison.

**Table 1:** Sequences and molecular properties of peptoids and comparator peptides.

Varient Category	Compound	Sequence	MW (Da)	HPLC elution	Net charge	CTLR
Basis	Peptoid 1 *	H-(*N*Lys-*N*spe-*N*spe)_4_-NH_2_	1819	65.1	4	0.33
Negative control	**1**_11mer_-*N*Leu	H-(*N*Lys-*N*Leu-*N*Leu)_3_-*N*Lys-*N*Leu-NH_2_	1321	51	4	0.36
Cationic charge	**1**-*N*Lys_5,11_ *	H-(*N*Lys-*N*spe-*N*spe-*N*Lys-*N*Lys-*N*spe)_2_-NH_2_	1753	51.2	6	0.5
	**1**-*N*btg	H-(*N*btg-*N*spe-*N*spe)_4_-NH_2_	1987	65	4	0.33
	**1**- *N*His_1,7_	H-(*N*His-*N*spe-*N*spe*-N*Lys-*N*spe-*N*spe)_2_-NH_2_	1767	62.5	2∼4**	0.17∼0.33
Length	**1**_13mer_ *	H-(*N*Lys-*N*spe-*N*spe)_4_-*N*Lys-NH_2_	1948	62.8	5	0.38
	**1**_11mer_ *	H-(*N*Lys-*N*spe-*N*spe)_3_-*N*Lys-*N*spe-NH_2_	1658	63.5	4	0.36
	**1**_9mer_ *	H-(*N*Lys-*N*spe-*N*spe)_3_-NH_2_	1368	60	3	0.33
Achiral	**1**_achiral_ *	H-(*N*Lys-*N*pm-*N*pm)_4_-NH_2_	1701	59.8	4	0.33
	**1**_achiral_-*N*spe_2_ *	H-*N*Lys-*N*spe-*N*pm-(*N*Lys-Npm-*N*pm)_3_-NH_2_	1721	60.8	4	0.33
	**1**_achiral_-*N*spe_2, 12_	H-*N*Lys-*N*spe-*N*pm-(*N*Lys-Npm-*N*pm)_2_-*N*Lys-*N*pm-*N*spe-NH_2_	1735	62	4	0.33
	**1**-*N*pm**_2,3,8,9_** *	H-(*N*Lys-*N*pm-*N*pm-*N*Lys-*N*spe-*N*spe)_2_-NH_2_	1763	63.3	4	0.33
Peptides	Pexiganan	GIGKFLKKAKKFGKAFVKILKK-NH_2_	2477	50.2	9	0.41
	LL 37	LLGDFFRKSKEKIGKEFKRIVQRIKDFLRNLVPRTES	4495	66	6	0.16

See Figure 1 for the structures of the peptoid monomers indicated in each sequence. HPLC elution is reported as the percentage of acetonitrile in water (% ACN) in the water/acetonitrile (0.1% trifluoroacetic acid) solvent in analytic HPLC. A linear water/acetonitrile (0.1% trifluoroacetic acid) gradient of 5%-95% acetonitrile over 30 min was run on a C18 column. Net charge indicates molecular charges at neutral pH. (“**”, *N*His has 10% probability to be charged around neutral pH, so the net charge of **1**- *N*His_1,7_ is between +2 to +4). CTLR stands for charge-to-length ratio, which is defined as the ratio of the total number of cationic residues to the total number of residues in each sequence. Peptoids labeled with asterisk have been reported previously to possess antimicrobial activities [23], [24], [25].

The activity and selectivity of these peptoids against cancer cells were evaluated *in vitro*. Their cytotoxicity was tested in three cancer cell lines, MCF-7 (human breast cancer), LNCaP (human prostate cancer), and OVCAR-3 (human ovarian cancer). To estimate the selectivity of these peptoids *in vitro*, hemolytic activities of some peptoids were cited from previously published work (Table 2 **column), and the MRC-5 cell line (human fibroblasts derived from fetal lung tissues) and primary dermal fibroblasts were also tested as normal cell controls. Briefly, cells were treated with peptoids diluted in culturing media for certain time periods, and then cell viability was measured via MTS assays. These peptoids as well as pexiganan and LL-37 were found to be more potent in media with low serum concentrations (data not shown), but results reported here are all tested in media supplemented with 10% FBS.

**Table 2:** Peptoid Cytotoxicity in cancer cell lines and control cells.

Variant Category	Compounds	LC_50_ (µM)					HC_10_ (µM) **
		MCF-7 (breast cancer)	LNCaP (prostate cancer)	OVCAR3 (ovarian cancer)	MRC-5 (fetal lung fibroblast)	Primary dermal fibroblast	
Basis	Peptoid 1 *	5	5	6	8	8	21
Negative control	**1**_11mer_-*N*Leu	>100	>100	>100	>100	>100	—
Cationic charge	**1**-*N*Lys_5,11_ *	31	25	37	41	34	>100
	**1**-*N*btg	5	8	8	10	8	—
	**1**- *N*His_1,7_	19	23	30	30	30	—
	**1**_13mer_ *	8	8	9	10	14	21
Length	**1**_11mer_ *	11	10	10	24	19	103
	**1**_9mer_ *	30	27	34	40	32	150
	**1**_achiral_ *	13	11	16	30	39	183
Achiral	**1**_achiral_-*N*spe_2_ *	13	11	16	22	36	160
	**1**_achiral_-*N*spe_2, 12_	12	10	16	21	32	—
	**1**-*N*pm**_2,3,8,9_** *	9	10	11	18	21	80
Peptides	Pexiganan	8	6	13	21	19	70
	LL 37	21	27	24	24	26	—

LC_50_ means lethal concentrations causing 50% of the cell death. The maximum peptoid and peptide concentration tested was 100 µM. Cell viability was measured via MTS assay in cells treated with compounds for 72 h, and LC_50_ of peptoids was derived from peptoids' viability curves. HC_10_ **, concentration causing 10% hemolysis of human red blood cells, all the numbers were cited from [25]. “—”, data not measured. Peptoids labeled with asterisk have been reported previously to possess antimicrobial activities [23], [24], [25].

**Figure 2:**
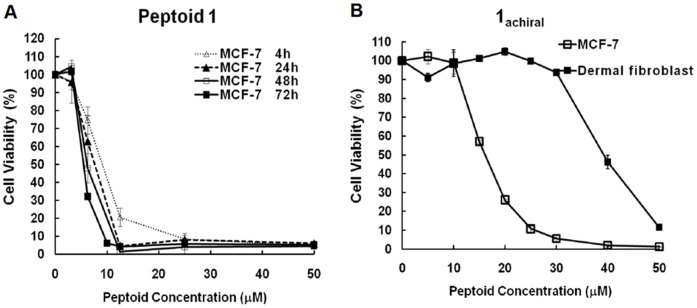
Cell viability curves of peptoids. A, cell viability of MCF-7 cells treated with peptoid 1 for different time periods. Cell viability was measured with MTS assays; B, cell viability curve of 1_achiral_ in MCF-7 and primary dermal fibroblast cells. 1_achiral_ was more toxic to MCF-7 cells.

These anticancer peptoids were found to exert fast killings in all tested cancer cell lines, and their activities were highly dependent on their structures and working concentrations. A majority of cell death occurred within only 4 h of peptoid treatments, and increased cytotoxicity was observed with longer treatments. The cytotoxicity curves of peptoid **1** in MCF-7 cells for different treatment times are shown in Figure 2A. Table 2 summarizes the compound activity for 72 h treatment to better correlate with cytotoxicity of apoptosis-inducing chemotherapeutics which are typically evaluated for 72 h treatment *in vitro*.

As shown in Table 2, LL-37, the non-selective antimicrobial peptide, showed little selectivity distinguishing cancer cell lines and normal control cells, while the clinically-relevant antimicrobial peptide, Pexiganan, exhibited modest *in vitro* selectivity towards cancer cell lines (indicated by higher LC_50_ in MRC-5 and primary dermal fibroblasts and higher HC_10_ against red blood cells than LC_50_ in cancer cell lines). We observed that the cytotoxicity of designed peptoids varied in different cancer cell lines, with LC_50_ in the low micromolar range. Some peptoids showed little selectivity, but several peptoids were found with modest *in vitro* selectivity towards cancer cells similar to Pexiganan, killing cancer cells efficiently while exhibiting less influence on MRC-5, primary dermal fibroblasts, and red blood cells in certain concentration ranges. How peptoid sequences could influence the cytotoxicity and selectivity will be discussed in the following structure-activity studies. The highest selectivity ratio (LC_50_ in primary dermal fibroblast divided by LC_50_ in cancer cells) we have observed for peptoids was ∼3 for **1**_achiral_ (Figure 2B). We grouped the peptoid hits into two categories: (1) Peptoid **1** is the most *potent* peptoid with good water solubility, ease of synthesis and relatively low hemolytic activity, though it has similar cytotoxicity against cancer cells and fibroblasts cultured *in vitro*; (2) Similar to Pexiganan, **1**_11mer_, **1**_achiral_ and **1**_achiral_-Nspe_2,12_ retain good potency and display modest *selectivity* in the *in vitro* screening.

### Structure-Activity Relationship Studies

#### Cationic, amphipathic structure

We intended to study how hydrophobic and cationic residues as well as the structural amphipathicity influenced peptoid potency and selectivity (Table 2). Knowing that aromatic side chains are critical for the biological activities of cationic, amphipathic peptoids [23], an 11-mer with *N*Leu as hydrophobic residues was designed as a negative control and was confirmed to be inactive, with LC_50_ above 100 µM in all the cells tested. Furthermore, hydrophobicity and structural amphipathicity were found to be important for biological activity. Reduced potency against mammalian cells was observed with **1**-*N*Lys_5,11_ which had reduced hydrophobicity with only 6 *N*spe residues per molecule and reduced amphipathicity with cationic residues present in hydrophobic faces [25]. Other cationic residues were also employed in peptoids. Guanidinium head groups have been previously reported to be critical for the cellular uptake of certain cell penetrating peptides [31]. Therefore, we synthesized **1**-*N*btg, or [*N*-(4-butylguanidine) glycine], by guanidinylating *N*Lys in peptoid **1**. The activity of **1**-*N*btg was similar to peptoid **1** without improvement on selectivity. Using histidines (pKa ∼6.1) as cationic residues has been reported to result in the pH-dependent activity of anticancer peptides with enhanced selectivity against tumor environments *in vivo* which are significantly more acidic than normal tissues [32]. **1**-*N*His_1,7_ was synthesized with *N*His replacing *N*Lys at position 1 and 7 in peptoid **1**, which would reduce peptoid cationic charges in neutral pH. Decreased cytotoxicity against both cancer cell lines and fibroblasts was observed with **1**-*N*His_1,7_. We did not observe a pH-dependent activity of this *N*His-*N*Lys hybrid peptoid *in vitro* (data not shown), consistent with the previous result of histidine-containing peptide which only showed enhanced selectivity *in vivo* [32]. **1**-*N*His_1,7_ could exhibit better selectivity *in vivo* yet needs to be further validated.

#### Chain length variants

The chain length is believed to influence not only the activity but also modes of action of cationic, amphipathic peptides [33], [34]. In a previous study, increasing the length beyond a 12mer, such as peptoid **1**_15mer_, [H-(*N*Lys-*N*spe-*N*spe)_5_-NH_2_], did not benefit antibacterial potency but resulted in substantially higher hemolytic activity, indicating that long chain lengths may lead to undesirable systemic toxicity of anticancer peptoids [23]. Thus we limited the chain length of peptoids to 13 residues at most, and studied **1**_9mer_ which has the same charge-to-length ratio (CTLR) as peptoid **1** at 0.33, **1**_11mer_ with CTLR at 0.36, and **1**_13mer_ with CTLR at 0.38 [25]. As shown in Table 2, the relatively low toxicity of 1_9mer_ against mammalian cells indicated that peptoids have to reach certain chain lengths to gain high potency. **1**_11mer_ was found to be more selective than peptoid **1** with reduced toxicity against MRC-5 and primary dermal fibroblasts and significantly reduced hemolytic activity, though its potency against cancer cells was slightly reduced as well. **1**_13mer_ was slightly less active than peptoid **1** yet without noticeable improvements on its selectivity.

#### Achiral monomer variants

The molecular chirality of peptoids is derived from the chirality of the side chains rather than that of the backbone [21]. **1**_achiral_ with less hydrophobic, achiral *N*pm residues lost helical signals in circular dichroism (CD) spectroscopy, which suggests a lack of stable secondary structure, and increased replacement of *N*spe with *N*pm in peptoid **1** decreased peptoid helical intensities in CD. The achiral monomer variants had significantly reduced hemolytic activities compared to peptoid **1** [25]. In our *in vitro* screening, **1**_achiral_, **1**_achiral_–*N*spe_2_, **1**_achiral_–*N*spe_2,12_, **1**-*N*pm_2,3,8,9_ all displayed better selectivity than peptoid **1** with reduced toxicity to MRC-5 cells and primary dermal fibroblasts. **1**_achiral_ was the most selective peptoid in this group with good potency against cancer cells (Figure 2B).

### Anticancer peptoids overcome multidrug resistance developed in cancer cells

**Figure 3:**
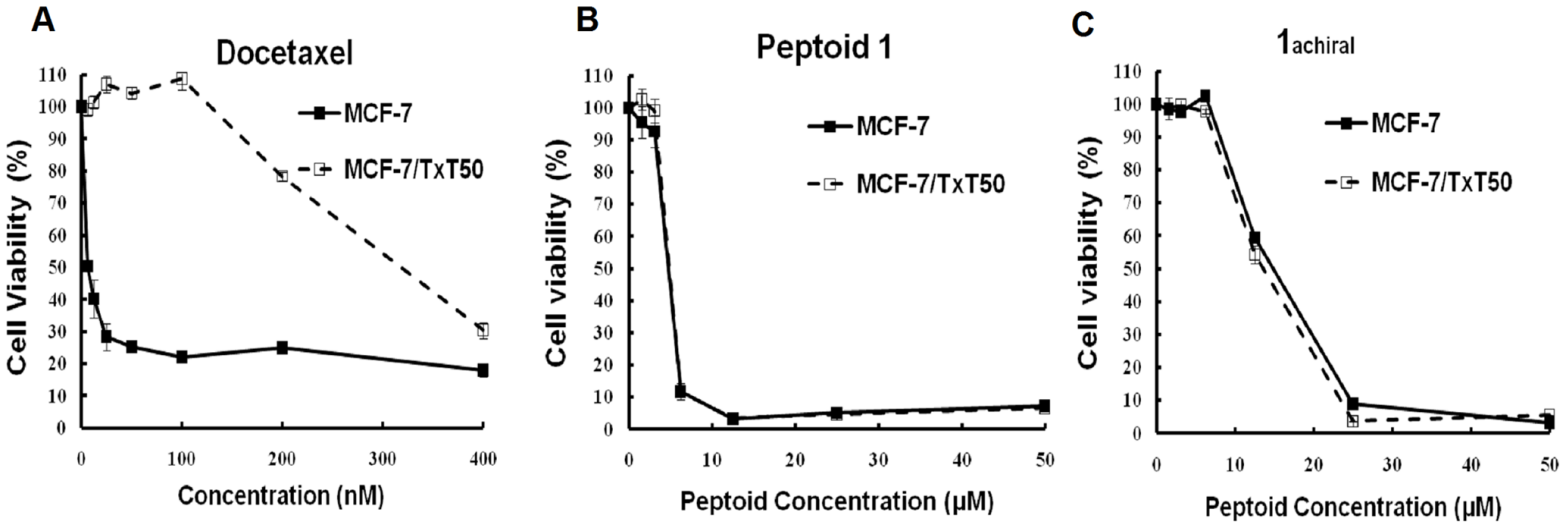
Activity of docetaxel and peptoids in MCF-7 and MCF-7/TxT50 cells. MCF-7/TxT50 cells were selected by docetaxel screening and are resistant due to overexpression of MDR1. Figures show the cell viability of MCF-7 and MCF-7/TxT50 cells treated with docetaxel (A), peptoid 1 (B) and 1_achiral_ (C) for 72 h. Cell viability was measured with MTS assays.

Several anticancer peptides, such as magainin 2 [12] and buforin IIb [13], [14], have been reported to overcome multidrug resistance developed in cancer cells. Interestingly, activities of our anticancer peptoids were also found to be unaffected by the multidrug resistance in cancer cells. The resistant MCF-7/TxT50 cell line which was selected by increasing exposure to docetaxel is resistant due to the high expression of the ABCB1/MDR1, P-glycoprotein [35]. As shown in Figure 3A, MCF-7/TxT50 cells were confirmed to be resistant to Docetaxel compared to wild-type MCF-7 cells. However, peptoid **1** and **1**_achiral_ had similar activities in both MCF-7 and the resistant MCF-7/TxT50 cells (Figure 3B-C), indicating a different mode of action of peptoids from the hydrophobic small molecule chemotherapeutic agents.

### Primary cytotoxicity of anticancer peptoids in cancer cells involves plasma membrane damage

**Figure 4:**
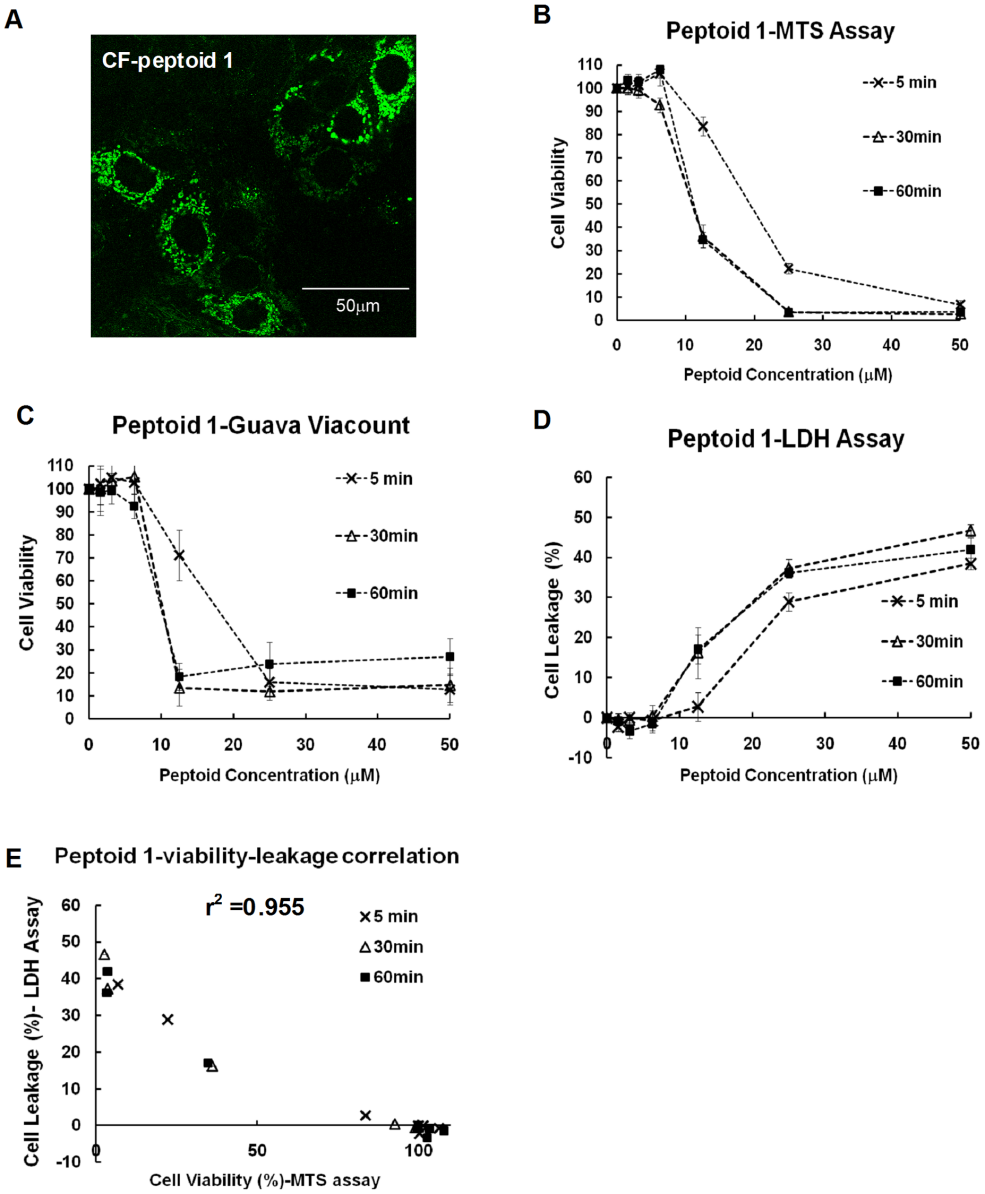
Killing mechanisms. A, live cell confocal images. MCF-7 cells were treated with 8 µM of CF-peptoid 1 for 1 h, and cells were imaged with ×63 oil lens. MCF-7 cells were treated with peptoids for indicated time and cell viability was measured with MTS assays after another 48 h incubation with fresh media (B), or measured via the Guava assay immediately after peptoid treatment (C), or quantified with LDH leakage immediately (D). E, correlation of LDH leakage and cell viability upon peptoid treatment measured with MTS assays, with r^2^ = 0.955.

These anticancer peptoids exert fast killing in cancer cells and are not affected by the multidrug resistance. To study how these peptoids interact with cells, we synthesized CF-peptoid **1** with fluorescent labeling on the N-terminus of peptoid **1**. Via live cell confocal imaging, peptoid **1** was found to penetrate into cells efficiently with a dot-like cytoplasmic distribution, even at low concentrations without causing any noticeable cytotoxicity (Figure 4A), indicating that these peptoids can interact with plasma membranes and translocate into cells. To evaluate if plasma membranes are damaged upon peptoid interactions and to determine how much membrane damage accounts for the cell death caused by peptoid **1**, cells were treated with peptoids at various concentrations for 5, 30 and 60 minutes. The cells were then either cultured in fresh media for another 48 h before testing via the MTS assay, which measures cell viability by tracking cellular metabolism (Figure 4B), or immediately tested via two cytotoxicity assays that evaluate plasma membrane intactness, the Guava Viacount assay (Figure 4C) and the lactate dehydrogenase (LDH) assay (Figure 4D). The Guava Viacount assay quantifies dead cells with a cell-impermeable DNA binding dye that can only stain cells with damaged membranes and cellular signals are measured by Guava flow cytometry. The LDH assay measures membrane damage by quantifying the leakage of the cytoplasmic enzyme LDH. Cell viability curves quantified by the Guava Viacount assay right after peptoid treatment were similar to those measured 48 h later with MTS assays, suggesting that plasma membranes were damaged upon peptoid treatments and most of the cell deaths caused by peptoids were due to plasma membrane damage. Moreover, in the LDH assay, a fast release of LDH into culturing media was also observed upon peptoid treatment. With LDH leakage caused by cell lysis solution as the 100% control, 50 µM of peptoid **1** (Figure 4D) and melittin (Figure S1) caused ∼50% of enzyme leakage. A linear correlation with r^2^ = 0.955 was observed between LDH leakage and cell viability quantified via the MTS assay (Figure 4E). Similar results were observed with melittin which is generally accepted to be lytic (Figure S1), further confirming the primary toxicity of peptoids being on plasma membranes. Moreover, typical apoptotic DNA ladders were not observed in cells after 24 h of peptoid **1** treatment (Figure S2). Taken together, these anticancer peptoids killed cancer cells primarily through plasma membrane damage. However, there could be intracellular targets as well, since peptoids can translocate into cells and are widely distributed in the cytoplasm, and we have observed increased cell death with longer peptoid treatment in some concentration ranges (Figure 2A).

### Peptoid 1 inhibits tumor growth in a clinically relevant orthotopic xenotransplantation model

**Figure 5:**
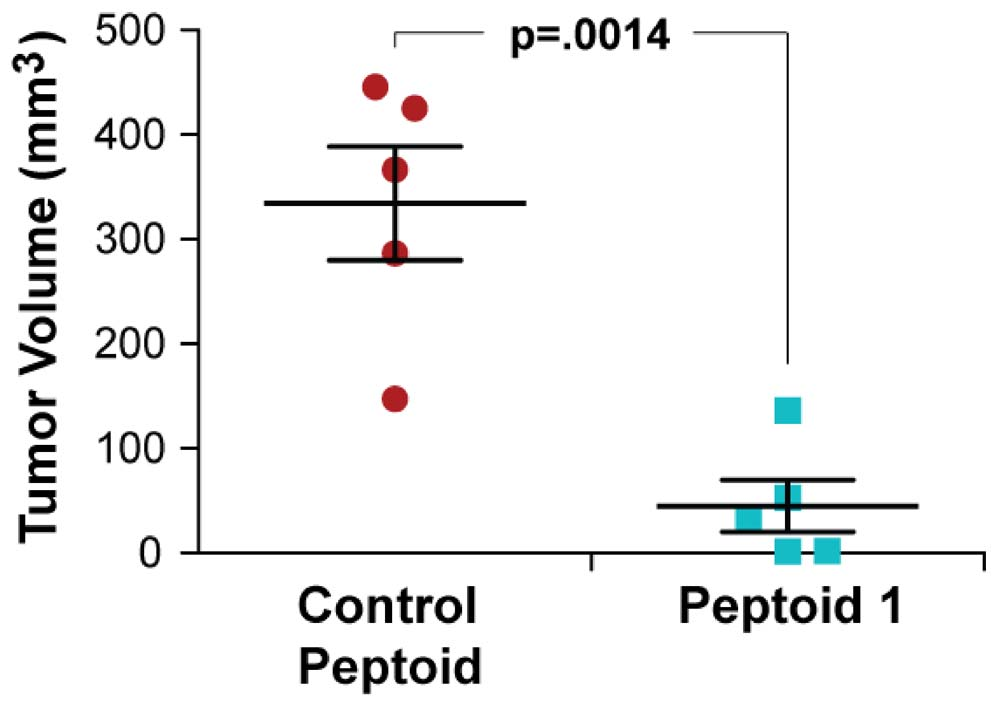
Reduction of tumor volumes in a human breast cancer xenograft mouse model treated with peptoid 1. Cancer cells (8×10^4^ cells) isolated from the second generation xenografts of human breast cancer tissues were implanted s.c. into the lower left mammary fat pads of 5–6-week-old immunocompromised NSG (NOD.Cg-Prkdc^scid^ Il2rg^tm1Wjl^/SzJ) female mice. Two weeks after cell implantation, 100 µl of peptoid 1 and control peptoids were injected into xenografts at 110 µM in PBS (∼1 mg/kg) three times a week, up to 8 weeks. Tumor sizes were measured by a caliper. P = 0.0014, *t* test.

As a preliminary study of the *in vivo* efficacy and safety of peptoids, the most potent peptoid, peptoid **1**, was evaluated in an orthotopic xenograft mouse model. Human breast cancer cells were implanted in the mammary fat pad of immunocompromised mice. After two weeks, peptoid **1** or the inactive negative control peptoid **1**_11mer_-*N*Leu was injected into the mammary fat pad at a dose of ∼1 mg/kg three times a week. Peptoid **1** significantly inhibited the tumor growth compared to the control peptoid (Figure 5). In addition, the applied dosages of peptoids did not cause any noticeable acute toxicity in mice. These results indicate that peptoid derivatives may be effective therapeutic agents for the treatment of human tumors, but their *in vivo* efficacy, toxicity, and proper local delivery methods will need to be thoroughly investigated in future.

## Discussion

Several cationic, amphipathic antimicrobial peptides or their derivatives have recently been reported with anticancer activity, displaying selective cytotoxicity against cancer cells *in vitro* and being effective in several *in vivo* xenograft models [3], [4], [7]. Moreover, a recent report indicated a naturally occurring anticancer role of a host defense peptide, cathelicidin, in natural killer cells' antitumor functions in mice, most strongly supported by the experiment that cathelicidin knockout (*Camp ^-/-^*) mice permitted faster tumor growth than wild type controls [36]. All these findings suggest that these cationic, amphipathic peptides, which are used by nature in host defense, represent a very interesting and promising group of candidates to be developed for anticancer applications.

In this study, we exploited the protease resistance and the propensity for helix formation of certain peptoids to develop stable anticancer peptidomimetics, expanding available monomer chemistry and capturing the cationic, amphipathic structural feature. Several anticancer peptoids were developed successfully with potent cytotoxicity toward a broad range of human cancer cell lines and their killing was not affected by multidrug resistance. Moreover, the most potent hit, peptoid **1**, greatly reduced tumor growth via intratumor injections in a human breast cancer xenograft mouse model without causing any obvious acute side effects in mice, suggesting the *in vivo* anticancer efficacy and good local tolerance of peptoids.

Our structure-activity studies revealed that it was critical for peptoids to have the cationic, amphipathic structure and to reach a certain chain length to obtain good potency against cancer cell lines. Peptoids needed to retain certain proportions of hydrophobic residues in their structures, namely a certain size of the hydrophobic arc in order to be potent. A typical ratio of cationic and hydrophobic residues per molecule was ∼1∶2 in peptoids with good potency. Increasing cationic residues (as in **1**-*N*Lys_5,11_ with 1∶1 cationic to hydrophobic ratio) or reducing cationic charges (as in **1**-*N*His_1,7_, with ∼90% of *N*His base in uncharged form around neutral pH) both weakened their activity. Moreover, using *N*btg which has a guanidinium head group instead of *N*Lys in peptoid **1** did not increase peptoid activity significantly. Instead, bulkier aromatic side chains in hydrophobic residues are found to be critical for peptoid potency. The cytotoxicity of **1**_11mer_-*N*Leu was low and was used as the inactive control. Taken together, the results with our peptoids support current explanations for the actions of cationic, amphipathic peptides: cationic residues select for anionic cellular membranes via electrostatic interactions; hydrophobic regions lead to membrane disruptions.

To evaluate peptoid selectivity *in vitro*, MRC-5 and primary dermal fibroblasts were tested as normal control cells, and hemolytic activities of several peptoids were also cited from previous work [25]. Though there are many reports about changed membrane properties of cells once they become cancerous [3], [7], [33], [37], it could be challenging to develop selective cationic, amphipathic peptides or peptoids to distinguish the slight differences. In our study, we found several peptoids with modest selectivity towards cancer cells, killing several cancer cell lines efficiently while showing little influence on normal control cells and red blood cells in some concentration ranges, such as achiral monomer peptoid variants with less stable secondary structures. The hemolytic activities of peptoids are generally quite low. These results prove the successful mimicry of anticancer peptides using non-natural peptoids.

The selectivity ratio of peptoids in our *in vitro* screening system was not strikingly high. Besides, given the membrane perturbation mechanism of these cationic, amphipathic molecules, the peptoids may only be suitable for certain anticancer applications, and local delivery or prodrug methods may mitigate any potential side effects. We understand that *in vitro* systems have limitations in mimicking *in vivo* environments; therefore, further investigation on the *in vivo* efficacy and toxicity as well as proper administration routes of these peptoids are required in a rigorous preclinical setting to establish their potential as cancer therapeutics.

There are several methods to further improve selectivity or reduce toxicity. First, we can engineer the peptoids at the molecular level by attaching “tumor homing” moieties to peptoids to enhance their accumulation in tumors and reduce their nonspecific interactions [38], [39], [40], or by developing peptoid-based prodrugs, the full activity of which need to be activated by tumor related enzymes [41], [42]. Second, systemic toxicity of peptoids can be tuned by the careful choice of delivery methods. Mitomycin C, a DNA crosslinking agent with antitumor antibiotic activity is administered as a single instillation within 6 hours of bladder tumor resection to minimize its toxicity and is proven to be effective in reducing recurrence [43]. The nonselective melittin was incorporated into nanocarriers with favorable pharmacokinetics, named “nanobees”, and was selectively accumulated in multiple tumor targets, dramatically reducing tumor growth without causing any apparent signs of toxicity [44]. Thus, a proper delivery method can be explored to further improve the *in vivo* behaviors of these peptoids.

Cationic, amphipathic peptides are generally believed to be membranolytic *in vitro*, accumulating on lipid membranes and subsequently disrupting the membrane structural integrity, either by forming pores in the membrane or acting like detergents and dissolving the membrane altogether [33]. We have observed membrane damage upon peptoid treatments, indicated by the Guava viacount assay and LDH assay, and there was a good correlation between immediate membrane damage and the cytotoxicity caused by peptoids. Interestingly, these peptoids can also penetrate into cells even at low concentrations. We even observed some co-localization of peptoids with mitochondria in the cytoplasm (data not shown). Therefore, we do not exclude potential intracellular targets of these peptoids, which is backed by the observation that longer peptoid treatments resulted in increased cell death at some concentration ranges. Our data support that these cationic, amphipathic peptoids interact with cells in a concentration dependent manner. At low concentrations, peptoids penetrate into cells in a way that does not disrupt plasma membranes or from which cells can recover, and they may disrupt intracellular organelles depending on their intracellular concentrations. At high concentrations, the way peptoids interact with plasma membranes causes membrane damage, which contributes to peptoids' observed, fast cytotoxicity.

Our preliminary *in vivo* experiment with peptoid **1** indicated promising anticancer potentials of these peptoids. Future research directions would include evaluating systemic toxicity of these peptoids in mouse models and testing their anticancer efficacy in clinical-relevant bladder cancer and ovarian cancer mouse models. This group of cationic, amphipathic peptoids, because of their broad spectrum of anticancer activity, unique modes of action, resistance to MDR and stability towards protease degradation, has a great potential to complement existing chemotherapy.

### Associated Content

Electronic Supplementary Information includes the evaluation data of melittin as a comparison in the mechanism study, the DNA ladder assay for peptoid treatment, and ESI-MS data of peptoids (Figure S1-3).

## Supporting Information

Figure S1 **Evaluation of melittin as a comparison.** MCF-7 cells were treated with melittin for indicated time and cell viability was measured with MTS assays after another 48 h incubation with fresh media (A), or measured via the Guava assay immediately after treatment (B), or quantified with LDH leakage immediately (C). D, correlation of LDH leakage and cell viability upon melittin treatment measured with MTS assays, with r^2^ = 0.726.
(TIF)

Figure S2 **DNA ladder assay.** MCF-7 cells were treated with control peptoid and peptoid 1 at the indicated concentrations for 24 h. Cells and floated cells were collected and combined for each sample. DNA was extracted using the apoptotic DNA ladder Kit (Roche), stained with GelStar (Lonza) and was run in 1% agarose gel. Experiments were done according to the kit, and the +DNA ladder control used the “lyophilized apoptotic U397 cells” sample provided in the kit as a positive control.
(TIF)

Figure S3 **ESI-MS data of peptoids.** Construct molecular weight (MW) and the corresponding peaks were indicated in the mass spectra. Previously reported peptoids in Table 1 are not listed here.
(TIF)
