# Supplementary figures and images for: Learning from Host-Defense Peptides: Cationic, Amphipathic Peptoids with Potent Anticancer Activity
Source: PLoS One. 2014 Feb 28;9(2):e90397. doi: 10.1371/journal.pone.0090397 (PMC3938723; doi:10.1371/journal.pone.0090397)

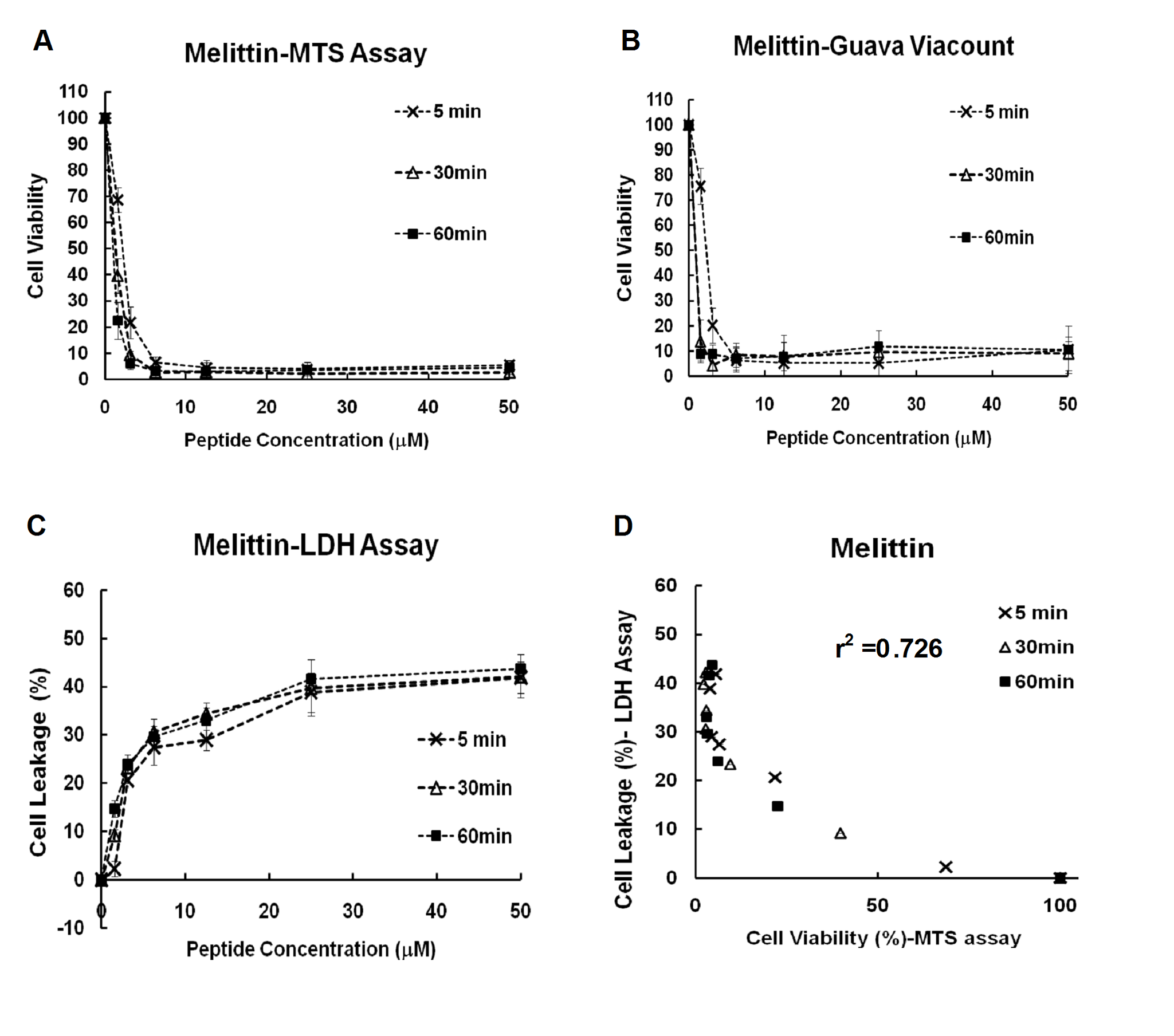

Supplement: Figure S1 — Evaluation of melittin as a comparison. MCF-7 cells were treated with melittin for indicated time and cell viability was measured with MTS assays after another 48 h incubation with fresh media (A), or measured via the Guava assay immediately after treatment (B), or quantified with LDH leakage immediately (C). D, correlation of LDH leakage and cell viability upon melittin treatment measured with MTS assays, with r2 = 0.726. (TIF) [file pone.0090397.s001.tif]

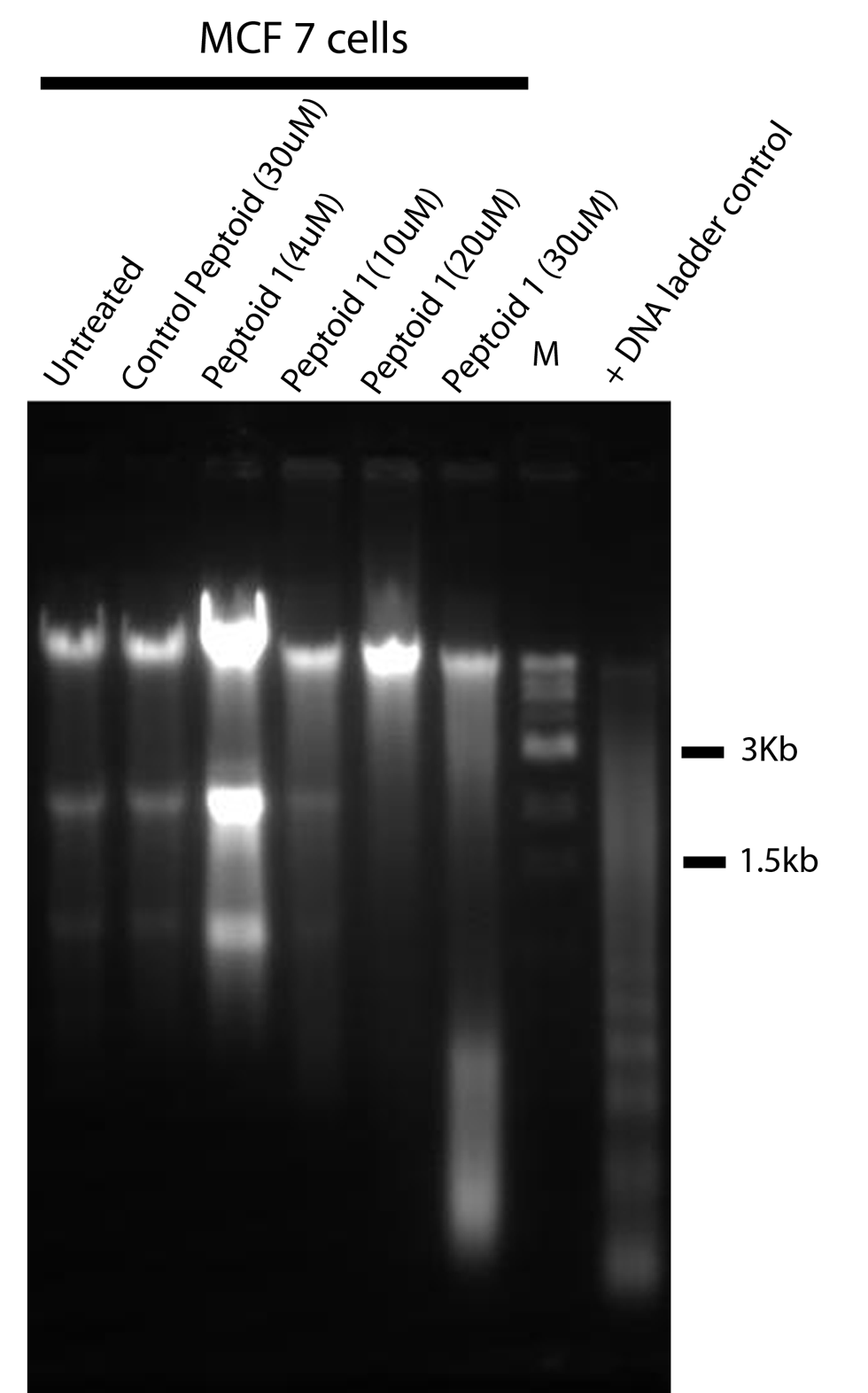

Supplement: Figure S2 — DNA ladder assay. MCF-7 cells were treated with control peptoid and peptoid 1 at the indicated concentrations for 24 h. Cells and floated cells were collected and combined for each sample. DNA was extracted using the apoptotic DNA ladder Kit (Roche), stained with GelStar (Lonza) and was run in 1% agarose gel. Experiments were done according to the kit, and the +DNA ladder control used the “lyophilized apoptotic U397 cells” sample provided in the kit as a positive control. (TIF) [file pone.0090397.s002.tif]

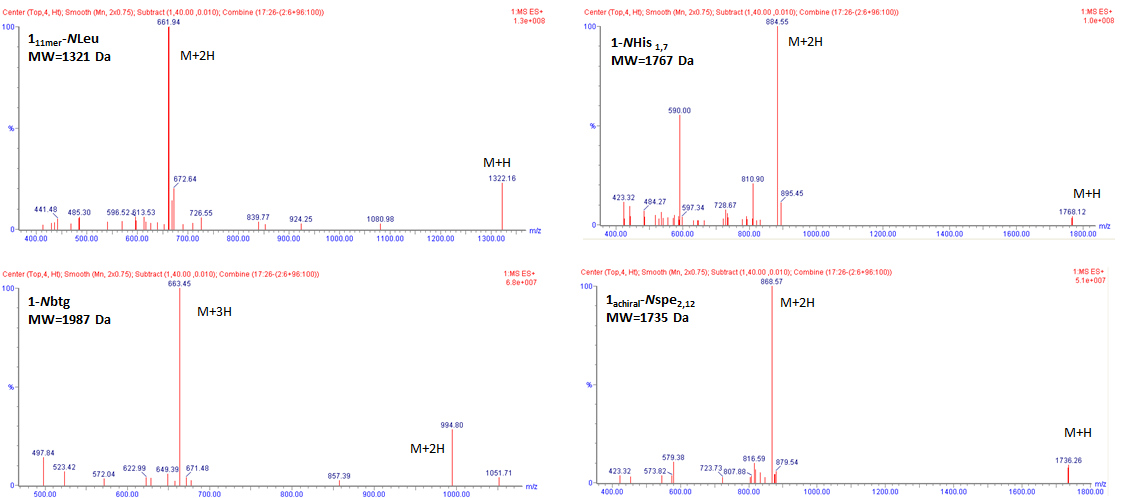

Supplement: Figure S3 — ESI-MS data of peptoids. Construct molecular weight (MW) and the corresponding peaks were indicated in the mass spectra. Previously reported peptoids in Table 1 are not listed here. (TIF) [file pone.0090397.s003.tif]
